# Paving the Way to Establish Protocols: Modeling and Predicting Mechanochemical Reactions

**DOI:** 10.1021/acs.jpclett.1c01472

**Published:** 2021-06-09

**Authors:** Eva Gil-González, Luis A. Pérez-Maqueda, Pedro E. Sánchez-Jiménez, Antonio Perejón

**Affiliations:** † Instituto de Ciencia de Materiales de Sevilla, Consejo Superior de Investigaciones Científicas−Universidad de Sevilla, Calle Américo Vespucio 49, Sevilla 41092, Spain; ‡ Departamento de Ingeniería Química, Universidad de Sevilla, Escuela Politécnica Superior, Calle Virgen de África, 7, Sevilla 41011, Spain; § Departamento de Química Inorgánica, Facultad de Química, Universidad de Sevilla, Sevilla 41012, Spain

## Abstract

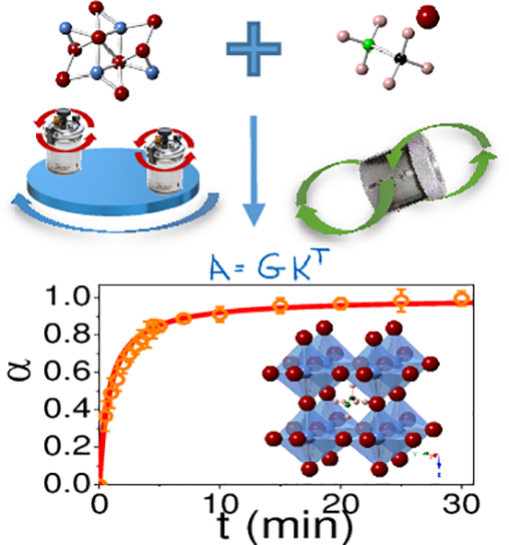

Parametrization of mechanochemical reactions, or relating the evolution of the reaction progress to the supplied input power, is required both to establish protocols and to gain insight into mechanochemical reactions. Thus, results could be compared, replicated, or scaled up even under different milling conditions, enlarging the domains of application of mechanochemistry. Here, we propose a procedure that allows the parametrization of mechanochemical reactions as a function of the supplied input power from the direct analysis of the milling experiments in a model-free approach, where neither the kinetic model function nor the rate constant equation are previously assumed. This procedure has been successfully tested with the mechanochemical reaction of CH_3_NH_3_PbCl_3_, enabling the possibility to make predictions regardless of the milling device as well as gaining insight into the reaction dynamic. This methodology can work for any other mechanical reaction and definitely paves the way to establish mechanochemistry as a standard synthetic procedure.

Mechanochemistry has undergone unprecedented growth in the past few decades, even though its use has expanded over history.^1^ This is due to its unique features, as mechanochemistry has proven to be a simple and highly flexible synthetic procedure. Different kinds of materials, either organic^2^ or inorganic,^3^ can be effectively prepared by basically grinding the starting materials together. Besides, mechanochemistry is capable of inducing high-temperature reactions at room temperature,^4^ surpassing certain solubility limit issues,^5,6^ obtaining metastable phases,^7,8^ or promoting the scaling up of reactions.^9^ Nevertheless, the most important feature of mechanochemistry is its ability to induce chemical reactions without the need of solvents, restricting the generation of toxic byproducts and, therefore, awarding it the label of a green synthetic procedure.^3,10,11^

In spite of the extended use of mechanochemistry, the understanding of its foundations is quite limited. How the mechanical energy is transferred into chemical energy and how the experimental parameters correlate with the underlying reaction mechanisms or the final properties of the resulting products are questions that remain elusive.^12^ This understanding is obviously of vital importance for reconciling the gap between the milling parameters, the reaction dynamic, and the resulting properties of the materials. Thus, some efforts, although still limited, have been made,^13^ such as the development of in situ characterization techniques for real-time monitoring of reactions,^14^ and as a way of example, we reference herein some studies devoted to investigate the effect of milling parameters on the kinetics of the reactions.^15−18^ Nevertheless, they contrast with most of the published works, which neglect the required attention to the milling conditions, making it extremely hard to establish milling protocols.

Thus, we propose a new methodology that allows a correlation of the underlying reaction mechanism with the milling parameters, specifically the applied input power calculated by simple analytical equations.^19^ This methodology is inspired by the nonparametric kinetic analysis (NPK),^20−22^ originally proposed for thermally activated processes, which is able to extract all of the kinetic information, i.e., the kinetic-triplet, to describe the process from a set of experimental data without any previous assumptions of the kinetic model describing the process.^23^ We prove that it is possible to predict experimental curves as long as the input power is known, independently from the milling device, which potentially can ease the reproducibility of mechanically induced reactions. This new methodology is tested with the mechanochemical production of the halide perovskite CH_3_NH_3_PbCl_3_ (MAPbCl_3_), a promising candidate for harvesting solar energy. Previous reports showed that these hybrid perovskites,^24^ even those with complex stoichiometry,^25,26^ can be effectively prepared by mechanochemistry, allowing a better control over the stoichiometry in comparison to wet chemical methods and leading to materials with improved properties. This proves, once again, the potential of mechanochemistry and the need to establish protocols.

The schematic process of the mechanosynthesis of MAPbCl_3_ is depicted in Scheme 1. The different milling conditions included in the kinetic analysis along with the total milling time, *t*_m_, required for the consumption of the raw materials and the applied input power, *P*, calculated according to Burgio’s equations^19^ are summarized in Table 1. See the Supporting Information (SI) for details about the experimental and kinematic calculations.

**Scheme 1:**
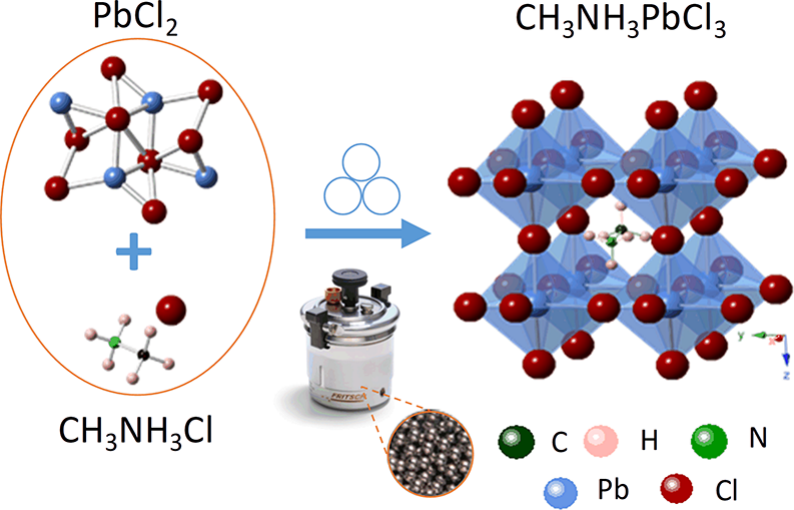
Schematic Representation of the Mechanosynthesis Process of CH_3_NH_3_PbCl_3_ (MAPbCl_3_) along with the Crystal Structure Diagrams

**Table 1:** Rotational Speed, Ball Diameter, Number of Balls, Total Milling Time, *t*_m_, Required for the Consumption of Raw Materials and Applied Input Power, *P*, Used in the Milling Conditions Included in the Kinetic Analysis

milling condition	rotational speed (rpm)	ball diameter (mm)	number of balls	total milling time, *t*_m_ (min)	input power, *P* (W)
1	250	15	9	370	1.2
2	250	10	30	360	1.3
3	300	15	9	170	2.1
4	400	15	9	60	4.9
5	500	15	9	30	9.6

To monitor the mechanically induced reaction of MAPbCl_3_, ex situ XRD data were collected at different milling times as shown in Figure 1a and Figures S1–S4. Even at a very short milling times (*t*_m_ = 1 min), there is already evidence of the formation of MAPbCl_3_ as confirmed by the XRD pattern and SEM image (Figure 1a,b). Morphologically, at this stage the powders are composed of a mixture of the starting materials (Figure S5) with some irregular hexagonal-like shaped particles of perovskite, which continue to increase with the milling time. EDX analysis confirmed that powders homogenized from the very beginning of the milling treatment, even for the lowest applied input power (Figure S6). Quantitative information about the phases presented during the milling treatment and, therefore, the degree of conversion, α, of raw materials into MAPbCl_3_ was carried out by Rietveld refinement as shown in Figure 1c and Figure S7, for the experimental condition 5, as a way of example. It is noteworthy that the mechanically induced reaction to form MAPbCl_3_ is not affected by the milling stops so that continuous and discontinuous millings lead to identical results provided that the total milling time is the same. These milling stops also prevent the overheating of the jars, in order to avoid undesired effects over the reaction kinetics. Thus, α versus milling time plots, α-*t*_m_,could be constructed for every milling condition as depicted in Figures 1d and 2, which also shows the morphology of powders at the end of each mechanochemical treatment. The powders are basically composed of highly aggregated clusters of irregular hexagonal-like shaped particles ranging from 400 nm to 1 μm approximately, characteristic of milled halide perovskites.^24,27^ In contrast to the hypothesis of Prochowicz et al., the morphology does not seems to be influenced by the milling conditions investigated here.^24^

**Figure 1:**
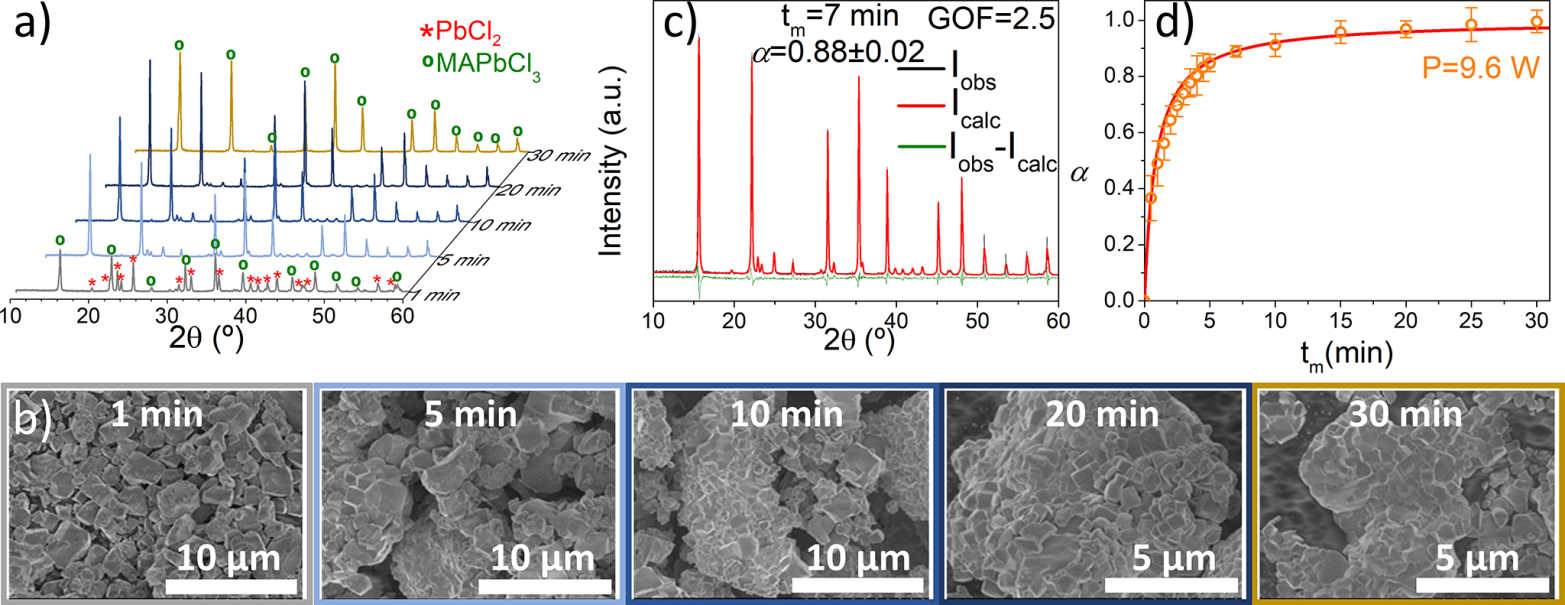
(a) Selected XRD-patterns, (b) morphological evolution, (c) Rietveld refinement (*t*_m_ = 7 min), and (d) degree of conversion, α, of starting materials as a function of the milling time, *t*_m_, under milling condition 5 (*P* = 9.6 W). The solid red line in part d corresponds to the simulated curve using the kinetic parameters obtained from the proposed method.

**Figure 2:**
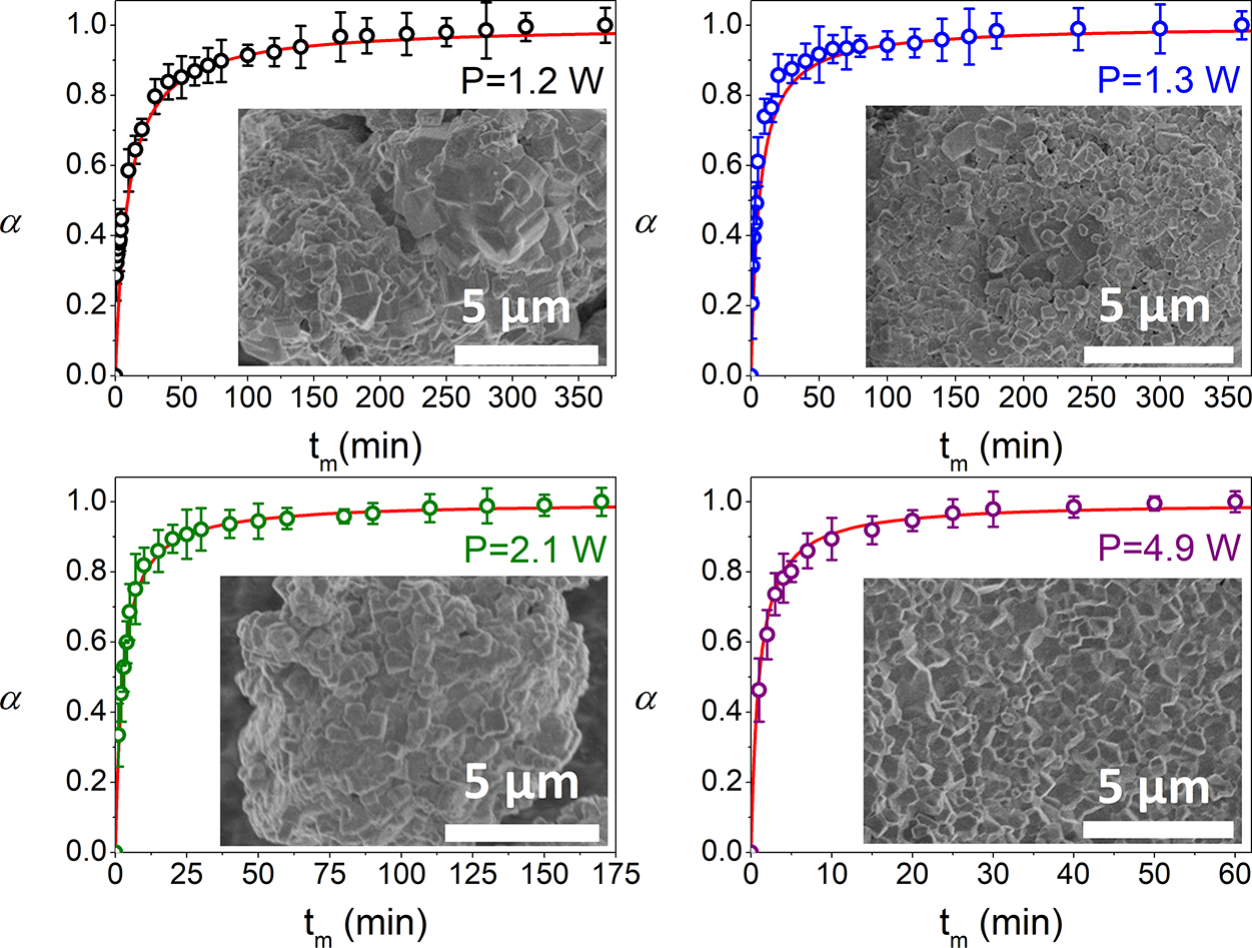
Experimental α-*t*_m_ plots, along with the microstructure (SEM micrographs) at the end of each mechanochemical treatment included in Table 1 (except condition 5 that is included in Figure 1). The solid red lines correspond to the simulated curves using the kinetic parameters obtained from the proposed method.

From a simple phenomenological analysis of the α–milling time plots, α–*t*_m_, the α values increase exponentially at short milling times, followed by continuously decelerating kinetics, which is probably limited by the mass transport of reactants while milling proceeds. This type of behavior is typical of mechanochemical reactions.^28^ Additionally, the higher the input power, the shorter the milling time required to complete the reaction. It is noteworthy that the input powers of conditions 1 and 2 are quite similar, 1.2 and 1.3 W, respectively, yielding similar milling times to complete the mechanochemical reaction. The main difference lies in the diameter and number of balls employed, which kinetically is translated in less impact energy and higher numbers of collisions from condition 1 to 2. Thus, reaction kinetics does not seem to be significantly influenced by the impact energy of each collision, which contrasts with the results of Fischer et al. for the cocrystallization of felodipine and imidazole.^17^ It might be related to the intrinsic nature of each chemical reaction instead. However, as it is characteristic of mechanically induced reactions, there should be a minimum threshold value of impact energy to activate the reaction of MAPbCl_3_.^28^ For this material, this threshold value is presumably quite low, as the chemical reaction of these kinds of perovskites can be even induced with just a mortar and pestle,^29^ and it is obviously surpassed in every mechanochemical treatment carried out here due to the machinery assistant.

As a further step from the above phenomenological description, an in-depth kinetic analysis was carried out as well. Note that the goals of a kinetic analysis are (1) parametrization of the reaction to be able to make predictions and (2) to extract information about the reaction kinetic model. Determining the kinetic parameters that properly describe a mechanochemical process is not a straightforward task, as the available procedures already published in the literature for solid-state reactions have been mostly developed for thermally activated process, and mechanochemical aspects are seldom considered.^23^ Nevertheless, some similarities can be established between both kinds of processes. In general, the reaction rate, dα/d*t*, of a solid-state process can be expressed as the product of two independent functions *k* and *f*(α):
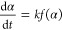
1where *k* is the rate constant, and *f*(α) is a function of the degree of conversion, α, corresponding to the kinetic model. In a thermally activated process, *k* is a function of temperature. Analogously, in a mechanochemical reaction, *k* should be a function of the supplied input power, *P*, as the process is mechanically driven. Indeed, previous models developed for mechanical processes show that the reaction rate is obviously a function of the supplied energy dose or the applied stress. For instance, Butyagin found that the rate constant of certain mechanochemical reactions follows an Arrhenius-type dependence with the expended energy.^30^ The supplied input power, *P*, is assumed to be constant during a mechanochemical treatment carried under a prefixed experimental condition. Although the applied mechanical field is not instantaneously released, this assumption is quite reasonable as a planetary ball mill reaches the preset rotational speed in just a few seconds, and as indicated, the reaction is not affected by the milling stops. Thus, *k*(*P*) is constant, and eq 1 can be integrated as follows:
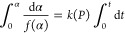
2

Equation 2 can also be expressed as
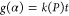
3where *g*(α) is the integral form of *f*(α). Table S1 shows some of the kinetic *f*(α) and *g*(α) functions corresponding to the most widely used kinetic models for solid-state processes.^31^

From isolating the milling time in eq 3
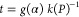
4it is shown that, similarly to the reaction rate, the milling time can also be expressed as the product of two independent functions: *g*(α) and the reverse of the reaction rate, *k*(*P*)^−1^, which account for the kinetic model that describes the process and its power supply dependence, respectively. The NPK method, originally proposed for thermally activated process by Serra, Nomen, and Sempere,^20,22^ is adapted here to mechanically activated processes. Its foundations rely on the fact that the reaction rate described in eq 1 can be discretized and organized as an *n* × *m* matrix whose rows and columns correspond to different degrees of conversion and temperatures, respectively.^20,22,32^ This methodology factorizes such matrix, providing two independent vectors that contain all the kinetic information about the process. One of those vectors holds the information about the kinetic model, while the other one carries the information about the reaction rate. Unlike most methodologies, the main advantage of this methodology is that there is no need to make any previous assumption about the kinetic model or rate constant function governing the processes.^32^

Extrapolating this concept to mechanically induced reactions and taking into account eq 4, the milling time can also be discretized in an *n* × *m* matrix, **A**, whose rows correspond to different degrees of conversion, from *α*_1_ to *α*_*n*_, and columns to different power input supply from *P*_1_ to *P*_*m*_. In other words, the rows are time at constant α varying the power supply, and the columns are time at constant *P* but different α. Thus, the matrix **A** is defined as:
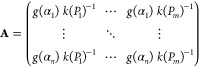
5

The matrix **A** can be factorized and written as two independent vectors:
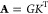
6where
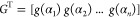
7
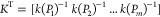
8

Those two vectors, eqs 7 and 8, contain all of the kinetic information about the process, avoiding the need of any previous assumption. On one hand, the kinetic model governing the process can be easily deduced by comparing the plot of the vector *G* (eq 7) versus α to those of the functions corresponding to the ideal models listed in Table S1.^31^ On the other hand, the nature of the rate constant *k*(*P*) can be obtained from the plot of the reverse of vector *K* (eq 8) against the power input supply *P.* The singular value decomposition (SVD) is used to decompose the matrix **A**, although another mathematical procedure can be used. This method factorizes **A** into three different matrices:
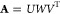
9

*U* and *V* are orthonormal matrices, containing information about the process dynamics and power supply dependence, respectively. *W* is a diagonal matrix whose elements are the singular values of the original matrix **A**. The relative weight of the columns of *U* and *V* is set by their corresponding singular values.^33^ If just the first singular value, *w*_11_, is significant, i.e., much bigger than the rest or different from zero, the matrix **A** can be expressed as
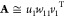
10where *u*_1_ and *v*_1_ are vectors corresponding to the first column of *U* and *V*, respectively. This means that vectors *u*_1_ and *v*_1_ are proportional to vectors *G* and *K* of eqs 7 and 8 (*G* ∝ *u*_1_ and *K* ∝ *v*_1_). Thus, the kinetic function governing the process can be directly extracted from *u*_1_, while the nature of the rate constant as a function of the input power supply can be obtained by fitting vector *v*_1_.

Results corresponding to the mechanochemical reaction to produce MAPbCl_3_ (included in Figures 1d and 2) are analyzed by the method described above. The obtained *u*_1_ and *v*_1_ vectors are depicted in Figure 3. On one hand, the plot of the *u*_1_ vector against α (Figure 3a) is very nicely fitted by a second-order deceleratory model F2, where the reaction rate is proportional to the concentration of reactants raised to power two. On the other hand, the function associated with the rate constant *k* can be extracted from vector *v*_1_ (Figure 3b). For thermally activated processes, the rate constant *k* has been traditionally described with an Arrhenius type equation, that, in a mechanically activated process, can be written as a function of the input power supply as follows:^30^
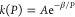
11where *A* is the pre-exponential factor, *P* is the power supply at which the mechanochemical treatment is being processed, and β is a parameter which is dimensionally equal to the power supply. Nevertheless, it may be also interesting to consider other non-Arrhenian equations (see Table S2), especially taking into account that this is a mechanically activated process.^34^Figure S8 shows the rate constant obtained from *v*_1_ as a function of the input power supply along with its fittings using the Arrhenius and several non-Arrhenian equations. All of them provide a good fit, probably due to the narrow range of applied power supply as explained by Šimon et al.^35^ For the sake of simplicity, the Arrhenius equation is chosen, where the inset of Figure 3b represents the Arrhenius-type dependence of the rate constant with the power supply along with the resulting kinetic parameters (β and *A* are calculated from the slope and intercept, respectively), also summarized in Table S3.

**Figure 3:**
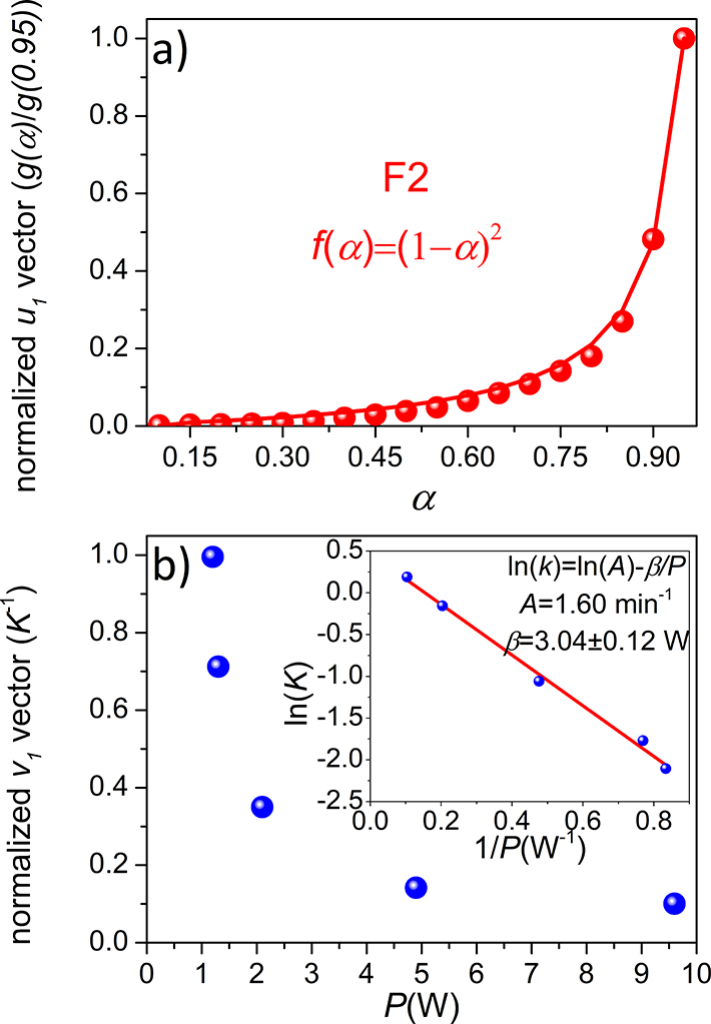
(a) Normalized *u*_1_ vector as a function of α, compared with the F2 kinetic model. (b) Normalized *v*_1_ vector as a function of the input power supply. The inset in part b corresponds to the Arrhenius plot: logarithm of *K* versus the reverse of the input power supply.

The most common procedure to evaluate the accuracy of the kinetic parameters consists of the simulation of the kinetic curves constructed with the obtained kinetic triplet (F2 kinetic function, *A* = 1.60 min^–1^ and β = 3.04 W). As shown in Figures 1d and 2, the simulated solid red lines are in very good agreement with the experimental data, proving the accuracy of the F2 model and the kinetic parameters obtained from the proposed methodology to describe the process. Thus, this procedure allows a reconciliation of the gap between the milling parameters and the underlying reaction dynamic in mechanochemistry, due to the soundness of the kinetic triplet obtained. It is worthy to highlight that, in a traditional kinetic analysis, the choice of the kinetic function is merely based on a statistical basis, which might return unreliable kinetic parameters.^36^ This uncertainty is overcome in the methodology proposed here, as neither the kinetic model nor parameters are previously assumed.

Nevertheless, the other ultimate consequence of a kinetic analysis is its capability to make predictions. Thus, the kinetic parameters were used to predict the behavior under the experimental conditions detailed in Table 2, which were not included in the analysis. It can be seen from Figure 4 that the experimental data are in very good agreement with the predictions, not only when a planetary ball mill is used (Figure 4a) but also when a shaker mill with a totally different motion is used (Figure 4b). How the input power supply was estimated for the SPEX mill is explained in the SI. Hence, unlike many other works that do not correlate the kinetics with the milling parameters or make predictions,^17,37−39^ it is shown here that the analysis of several kinetic curves by this kinetic procedure allows the description and even the prediction of mechanically induced reactions as a function of the applied input power supply regardless of the milling device. This can be an extremely useful tool to reproduce results from lab to lab or for the scaling up of processes.

**Table 2:** Milling Device, Rotational Speed or Frequency, Ball Diameter, Number of Balls, and Applied Input Power, *P*, Used in the Milling Conditions Not Included in the Kinetic Analysis^a^

milling device	rotational speed (rpm) or frequency (Hz)	ball diameter (mm)	number of balls	input power *P* (W)
planetary ball mill	700 (rpm)	15	9	26.3
SPEX mill	50 (Hz)	10	11	6.8

a These experimental conditions were used to validate the soundness and capability of the obtained kinetic parameters to make predictions. See the SI for further information about the experimental milling conditions.

**Figure 4:**
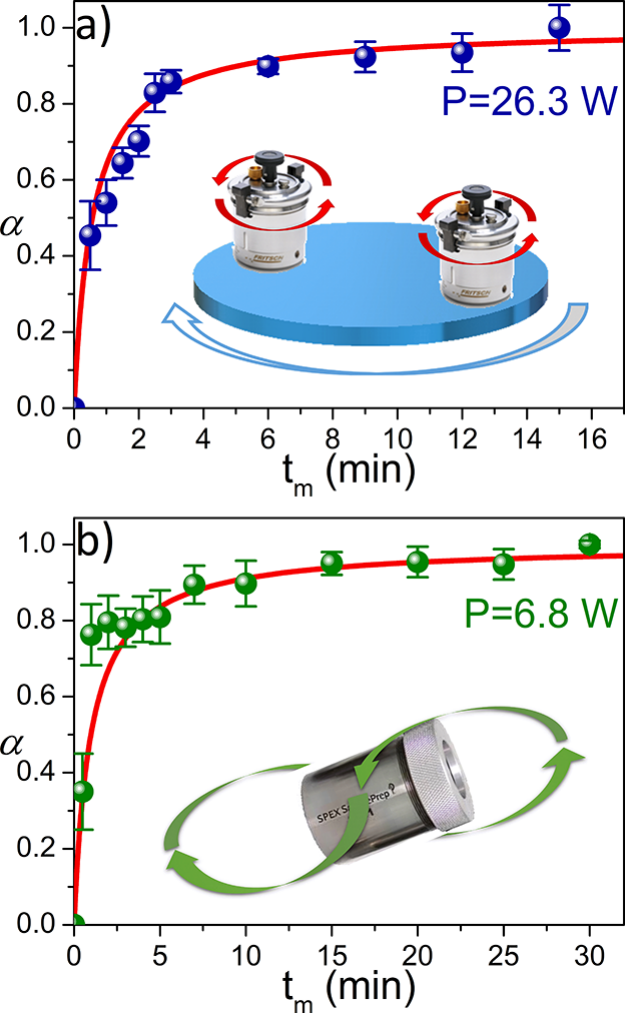
Experimental (dots) and predicted α–*t*_m_ curves (solid lines) using the kinetic triplet obtained from the proposed analysis (Table S3) for the mechanochemical reaction to produce MAPbCl_3_ in (a) a planetary ball mill and (b) a shaker SPEX mill. The specific experimental conditions are detailed in Table 2, while the corresponding XRD patterns are represented in Figures S9 and S10, respectively.

In conclusion, we have shown that the proposed kinetic analysis applied to the mechanochemical reaction between PbCl_2_ and MACl to produce MAPbCl_3_ allows (1) parametrization and prediction of reactions as a function of the input power supply independently from the milling device and (2) insight into the underlying reaction mechanism. This entails the reconciliation between the milling parameters and the reaction dynamics, which is extremely important to convert mechanochemistry into a standard synthetic procedure in chemistry. It is basically possible due to the reliability of the kinetic parameters and function obtained from the kinetic method developed here, as neither the kinetic function nor the rate constant equation are previously assumed. This methodology can work for other mechanically induced reactions, and we truly believe that it paves the way to establish protocols in mechanochemistry and expands its domains of application.

## Supporting Information Available

Experimental section; kinematic analysis; kinetic model and rate constant functions; XRD patterns, SEM images, and Rietveld refinements as a function of the milling time; and rate constant fittings using non-Arrhenian equations (PDF)
