# Supplementary material for: Paving the Way to Establish Protocols: Modeling and Predicting Mechanochemical Reactions
Source: J Phys Chem Lett. 2021 Jun 9;12(23):5540–6. doi: 10.1021/acs.jpclett.1c01472 (PMC8280717; doi:10.1021/acs.jpclett.1c01472)
Supplement: Supplementary file 1 — jz1c01472_si_001.pdf [file jz1c01472_si_001.pdf]

Supporting Information

Paving the Way to Establish Protocols: Modeling and Predicting Mechanochemical Reactions

Eva Gil-González[a,b*], Luis A. Pérez-Maqueda[a*], Pedro E. Sánchez-Jiménez[a], Antonio Perejón[a,c]

[a]*Instituto de Ciencia de Materiales de Sevilla, Consejo Superior de Investigaciones Científicas−Universidad de Sevilla. Calle Américo Vespucio 49, Sevilla 41092, Spain*

[b] *Departamento de Ingeniería Química, Universidad de Sevilla, Escuela Politécnica Superior, Calle Virgen de África, 7, Sevilla, 41011, Spain*

[c] *Departamento de Química Inorgánica, Facultad de Química, Universidad de Sevilla, Sevilla 41071, Spain*

*Corresponding authors: Eva Gil-González: eva.gil@icmse.csic.es, Luis A. Pérez-Maqueda: maqueda@cica.es.

List of Supplementary Content

1. Experimental 3

1.1 Material synthesis and characterization 3

2. Kinematic Analysis 3

2.1. Kinematic Analysis on planetary ball mills 3

2.2. Kinematic Analysis on a SPEX mill 4

3. Tables 6

4. Figures 7

5. References 15

List of Supplementary Tables

Table S 1. Kinetic $f(\alpha)$ and $g(\alpha)$ functions corresponding to the most used kinetic models...... 6

Table S 2. Non-Arrhenian equations for the rate constant as a function of the input power supply, *P*. *A* is the pre-exponential factor and *C*,*σ*,*a* and *b* are empirical factors. 6

Table S 3. Kinetic triplet obtained from the proposed analysis for the mechanochemical reaction to produce $MAPCl_3$. 7

List of Supplementary Figures and Legends

Figure S 1. Selected XRD patterns as a function of the milling time for experimental Condition 1 included in Table 1. 8

Figure S 2. Selected XRD patterns as a function of the milling time for experimental Condition 2 included in Table 1. 8

Figure S 3. Selected XRD patterns as a function of the milling time for experimental Condition 3 included in Table 1. 9

Figure S 4. Selected XRD patterns as a function of the milling time for experimental Condition 4 included in Table 1. 9

Figure S 5. SEM micrographs of the starting materials CH_3NH_3Cl (MACl) and $PbCl_2$. 10

Figure S 6. SEM micrographs (backscattered electrons) and chemical mappings taken at the beginning (t_m = 1 min) and at the end (t_m = 370 min) of the milling process under experimental condition 1 (P = 1.2 W). The distribution of the chemical compounds is homogenous even at very short milling time (t_m = 1 min). 11

Figure S 7. Rietveld Refinements of selected XRD patterns at different milling times for experimental Condition 5 (P = 9.6 W). 12

Figure S 8. Rate constant obtained from the v_1 vector as a function of the input power supply and its fittings using the Arrhenius equation as well as several Non-Arrhenian equations (Kooij, VFT or WLF and van't Hoff). 13

Figure S 9. Selected XRD patterns as a function of the milling time for the milling experiment of Table 2, carried out in a planetary ball mill. 13

Figure S 10. Selected XRD patterns as a function of the milling time for the milling experiment of Table 2, carried out in a SPEX mill. 14

1. Experimental

1.1 Material synthesis and characterization

Stoichiometric amounts of $PbCl_2$ (Sigma-Aldrich, 203572, 99.999% in purity) and CH_3NH_2HCl (MACl) (Sigma-Aldrich, 3588822, 99 % in purity) powders were charged in jars and milled in a planetary Micro Mill Pulverisette 7 Premium Line (Fritsch, Germany) or a shaker SPEX 8000M Mixer/Mill (SPEX SamplePrep, USA). A jar of 46.55 mm of diameter and 44.7 mm of height was employed in the planetary mill whereas one of 38 mm of diameter and 57 mm of height was used in the shaker mill. Hardened steel balls were used as grinding media and the ball to powder mass ratio (BPR) employed in each milling treatment was 20:1. When applicable, the maximum period of continuous milling in each mechanochemical treatment was set at 10 min, followed by 2 min of resting time in order to avoid the overheating of the jars. During the whole process, the powders were manipulated in a globe box filled with nitrogen, including the loading of the jars, to avoid the degradation of the materials.

In order to obtain the α-milling time curves, α-t_m, small amounts of samples were routinely collected from the jars at different milling times and analyzed by Rietveld Refinements. Thus, X-Ray diffraction (XRD) patterns were collected in a Rigaku Miniflex diffractometer (CuKα radiation, $\lambda = 0.15406$ nm) working at 40 kV and 15 mA. Measurements were taken within a 2θ range from 10º to 60º, with a 0.02 º step and a scan speed of 0.24 seconds per step. Rietveld Refinements were carried out with X'Pert HighScore Plus software.

The morphology and chemical composition of the powders were examined by Scanning Electron Microscopy (SEM) in a Hitachi S-4800 SEM-FEG, equipped with an energy dispersive X-ray spectrometer (EDX), Quantax Bruker. The field-emission gun was operated at 2 kV to obtain the micrographs, whereas for the EDX analysis and compositional chemical mappings, it was operated at 20 kV.

2. Kinematic Analysis

2.1. Kinematic Analysis on planetary ball mills

The power input, P, of each mechanical treatment was calculated according to the theoretical-empirical equation proposed by Burgio et al.[1] This model has been shown to be extremely reliable to assess the energy transferring in planetary ball mills,[2] even though it is based on several assumptions that serve to simplify and ease the calculations. For instance, Bugio's model is a "hit model", as the energy is only considered to be released by collisions between balls and the walls, being other phenomena such as friction and temperature rise (minimized by the

milling stops) negligible. The balls are assumed to move solidal with the jar without rolling and sliding on the inner wall of the jar and, therefore, the power input, P, can be calculated as:

$$P = \frac{1}{2} m_b W_p^2 \left[\left(\frac{W_v}{W_p}\right)^2 \left(\frac{D_v - d_b}{2}\right)^2 \left(1 - 2\frac{W_v}{W_p}\right) - 2R_p \left(\frac{W_v}{W_p}\right) \left(\frac{D_v - d_b}{2}\right) - \left(\frac{W_v}{W_p}\right)^2 \left(\frac{D_v - d_b}{2}\right)^2 \right] \varphi_b N f \quad (1)$$

where m_b and d_b are the mass and the diameter of a ball, D_v the diameter of the jars, R_p the distances from the center of the mill to the center of the vial, W_p and W_v are the angular velocity of the supporting disk and the jars, respectively, N is the number of balls in the jars, φ_b the hindering factor, as more than one ball contributes simultaneously to the milling process and f is the frequency at which balls are launched against the opposite wall of the jars. f can be calculated considering the following formula:

$$f = K \frac{W_p - W_v}{2\pi} \quad (2)$$

where K is a an empirical constant that depends on the mill geometry and diameter of the employed balls.

As it has been pointed out, this model provides the energy or power supplied from the collisions balls-walls, where actually just a fraction of it, particularly the one from the balls to the powders trapped during collisions is truly contributing to the mechanochemical event. Nevertheless, it is worth emphasizing that the model is extremely reliable to describe the energy transferring in planetary ball mills and it has been even meticulously validated by external authors.[2,3]

2.2. Kinematic Analysis on a SPEX mill

Note that the aim of this sections is to roughly calculate the input power supply on a SPEX mill. The motion of this type of mill is quite complex and many papers devoted to calculate its dynamic and motion by numeral modeling and simulation can be found in the literature.[4–7] However, this is out of the scope of this article, as the aim of this section is to roughly estimate the input power supply by a SPEX mill with simple analytical equations. Of course, due to the complexity, this is just a mere approximation and in order to make more precise predictions a more sophisticated calculation procedure should be employed like those referenced above. We have estimated the input power supply considering a linear trajectory of the ball and the classical equation of motion:

$$\mathrm{P} = \frac{1}{2} m_b v^2 \varphi_b N f \quad (3)$$

where m_b is the mass of a ball, v the velocity, φ_b the hindering factor, as more than one ball contributes simultaneously to the milling process, N is the number of balls in the jar, and f is the

frequency of impact. The amplitude of movement in a SPEX is 5.7 cm and the number of oscillations per unit of time depends on the frequency of the power source. It has been calculated by stroboscopic techniques that a 60 Hz power source results in about 17 cycles/s, whereas at 50 Hz results in approximately 14.2 cycles/s.[8]

3. Tables

Table S 1. Kinetic $f(\alpha)$ and $g(\alpha)$ functions corresponding to the most used kinetic models.

Mechanism	Symbol	$f(\alpha)$	$g(\alpha)$
First order	F1	$(1-\alpha)$	$-ln(1-\alpha)$
Second order	F2	$(1-\alpha)^2$	$\left[\frac{1}{1-\alpha}\right]-1$
Third-order	F3	$(1-\alpha)^3$	$\frac{1}{2}[(1-\alpha)^{-2}-1]$
Phase boundary controlled reaction (contracting area)	R2	$(1-\alpha)^{1/2}$	$2[1-(1-\alpha)^{1/2}]$
Phase boundary controlled reaction (contracting volume)	R3	$(1-\alpha)^{2/3}$	$3[1-(1-\alpha)^{1/3}]$
Random nucleation and growth of nuclei through different nucleation and nucleus growth models (Avrami-Erofeev eq. n ≠ 1)	An	$n(1-\alpha)[-\ln(1-\alpha)]^{1-\frac{1}{n}}$	$[-ln(1-\alpha)]^{\frac{1}{n}}$
Two-dimensional diffusion	D2	$1/[-\ln(1-\alpha)]$	$(1-\alpha)\ln(1-\alpha)+\alpha$
Three-dimensional diffusion (Jander eq.)	D3	$3(1-\alpha)^{2/3}/\left[2(1-(1-\alpha)^{\frac{1}{3}})\right]$	$\left[(1-(1-\alpha)^{\frac{1}{3}})\right]^2$
Three-dimensional diffusion (Ginstling-Brounshtein eq.)	D4	$3/[2((1-\alpha)^{-1/3}-1)]$	$\left(1-2\frac{\alpha}{3}\right)-(1-\alpha)^{\frac{2}{3}}$

Table S 2. Non-Arrhenian equations for the rate constant as a function of the input power supply, *P*. A is the pre-exponential factor and *C*,*σ*,*a* and *b* are empirical factors.

$k(P)=Ae^{\sigma P}$	Berthelot-Hood
$k(P)=AP^{C}$	Harecourt-Esson
$k(P)=Ae^{-\frac{\beta}{P}}$	Arrhenius
$k(P)=AP^{C}e^{-\frac{\beta}{P}}$	Kooij
$k(P)=AP^{C}e^{\sigma P}e^{-\frac{\beta}{P}}$	van't Hoff
$k(P)=Ae^{-\frac{a}{P+b}}$	Vogel-Fulcher-Tammann (VFT) or Williams-Landel-Ferry (WLF)

Table S 3. Kinetic triplet obtained from the proposed analysis for the mechanochemical reaction to produce $MAPCl_3$.

Kinetic model	F2:= $(1-\alpha)^2$
A (min^{-1})	1.60
β (W)	3.04 ± 0.12

4. Figures

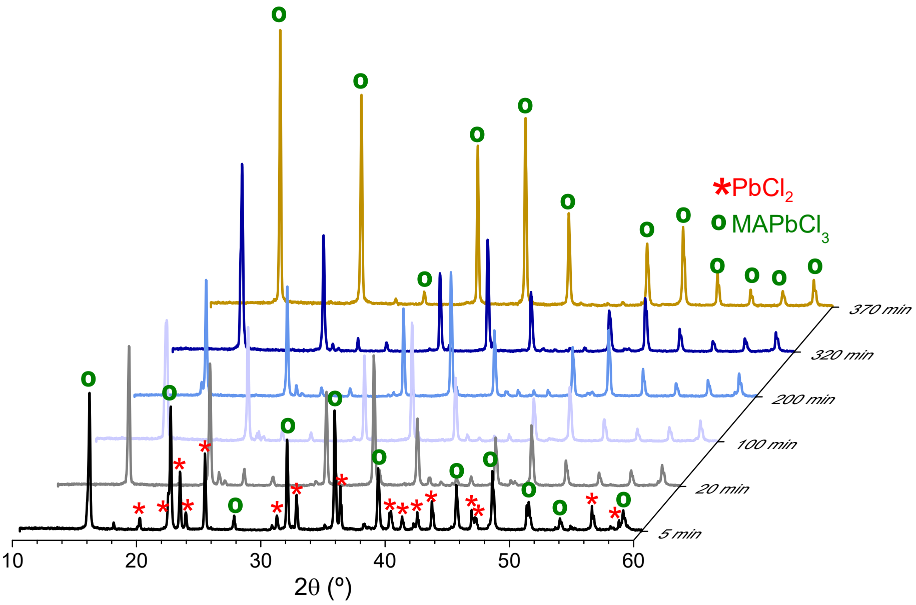

Figure S 1. Selected XRD patterns as a function of the milling time for experimental Condition 1 included in Table 1.

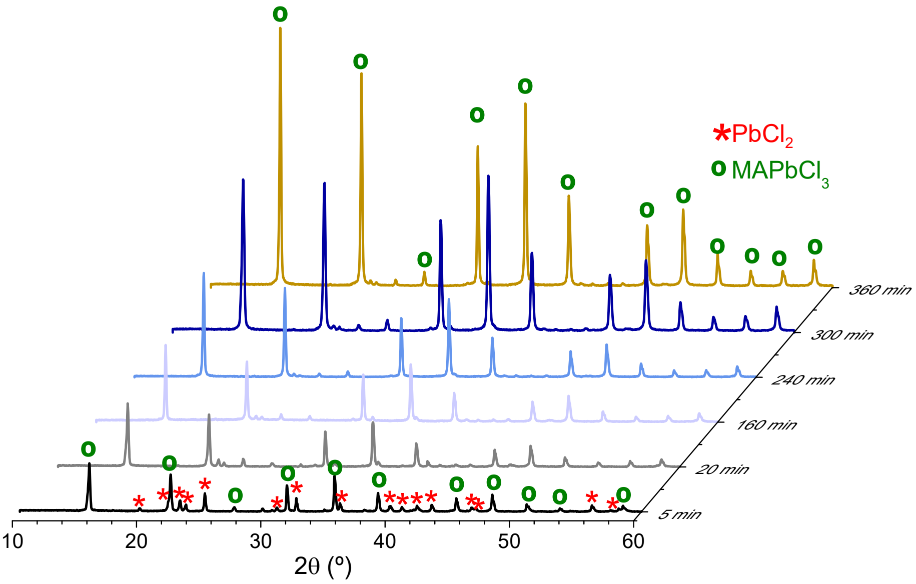

Figure S 2. Selected XRD patterns as a function of the milling time for experimental Condition 2 included in Table 1.

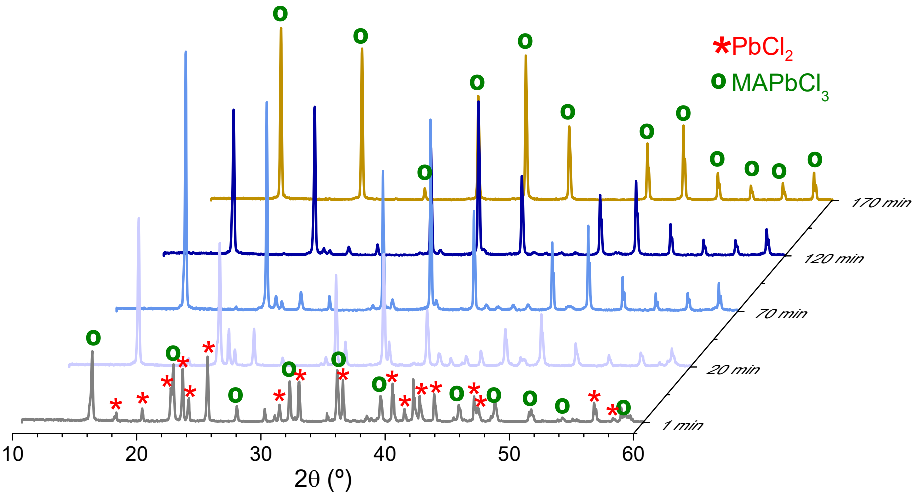

Figure S 3. Selected XRD patterns as a function of the milling time for experimental Condition 3 included in Table 1.

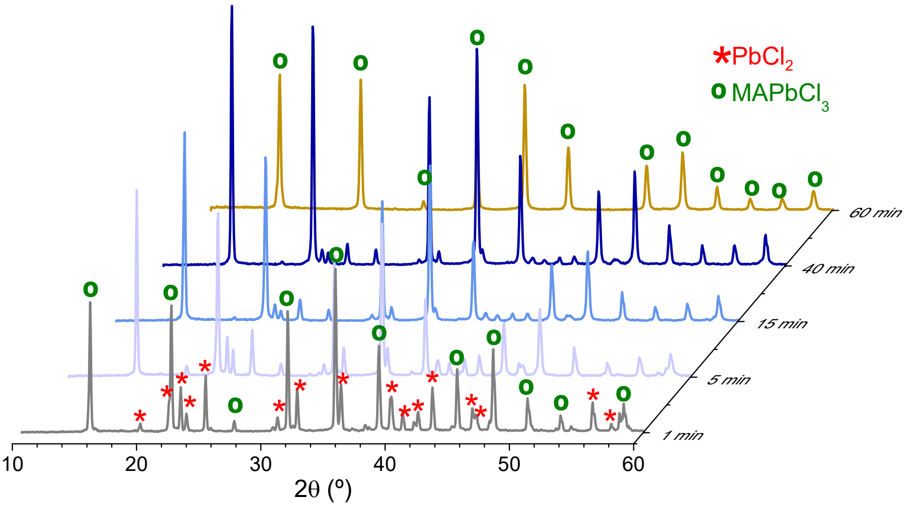

Figure S 4. Selected XRD patterns as a function of the milling time for experimental Condition 4 included in Table 1.

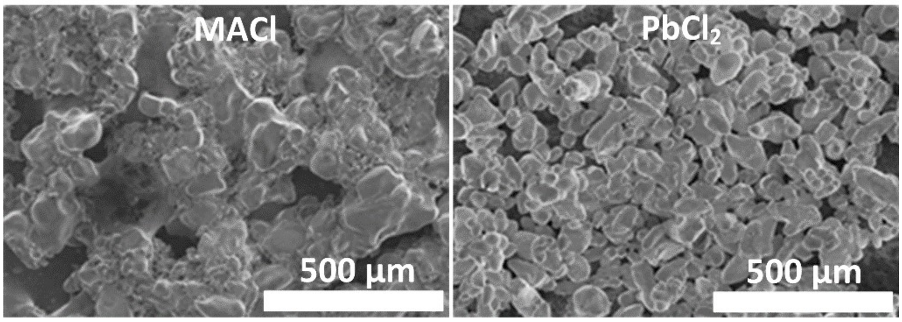

Figure S 5. SEM micrographs of the starting materials CH_3NH_3Cl (MACl) and $PbCl_2$.

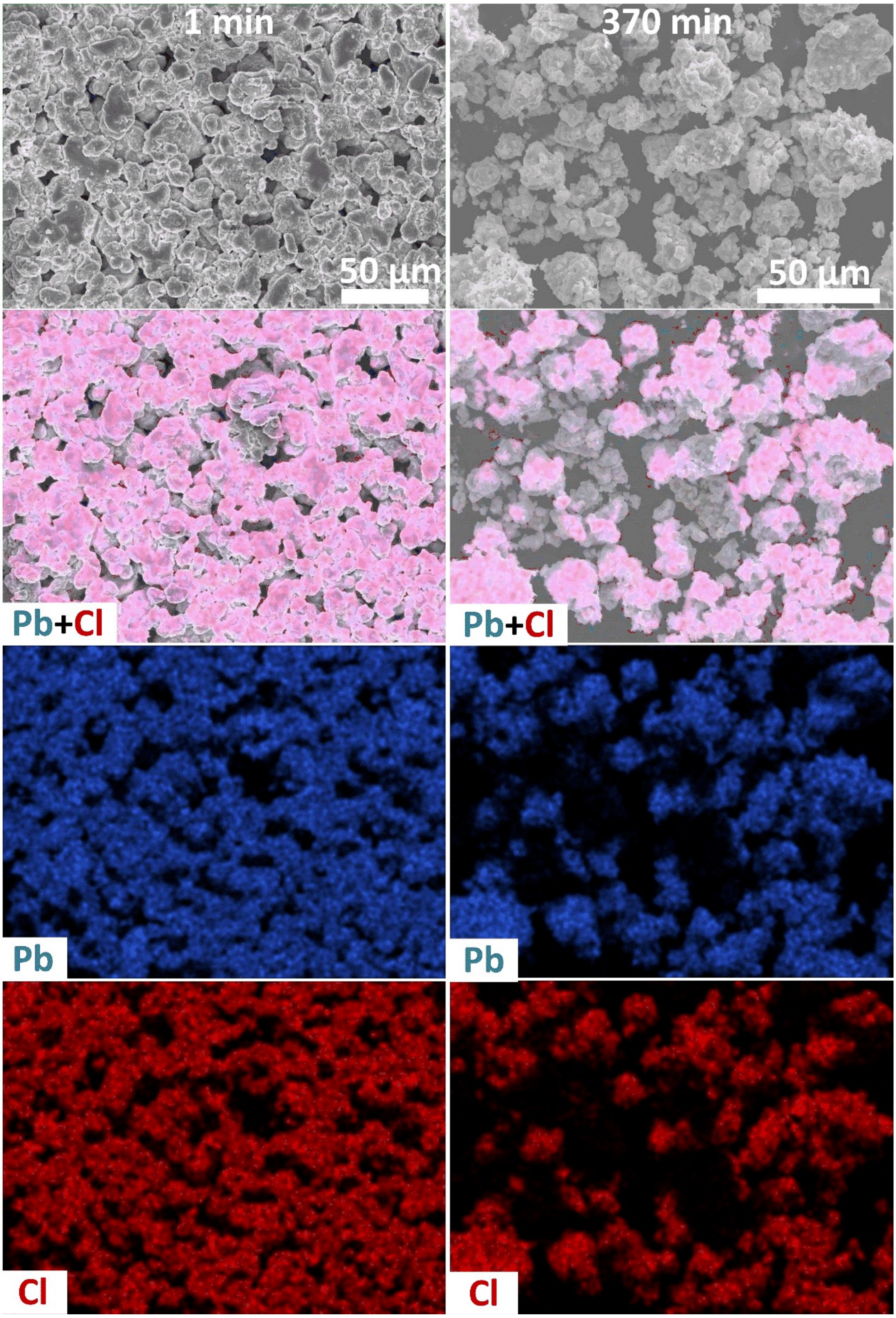

Figure S 6. SEM micrographs (backscattered electrons) and chemical mappings taken at the beginning (t_m = 1 min) and at the end (t_m = 370 min) of the milling process under experimental condition 1 (P = 1.2 W). The distribution of the chemical compounds is homogenous even at very short milling time (t_m = 1 min).

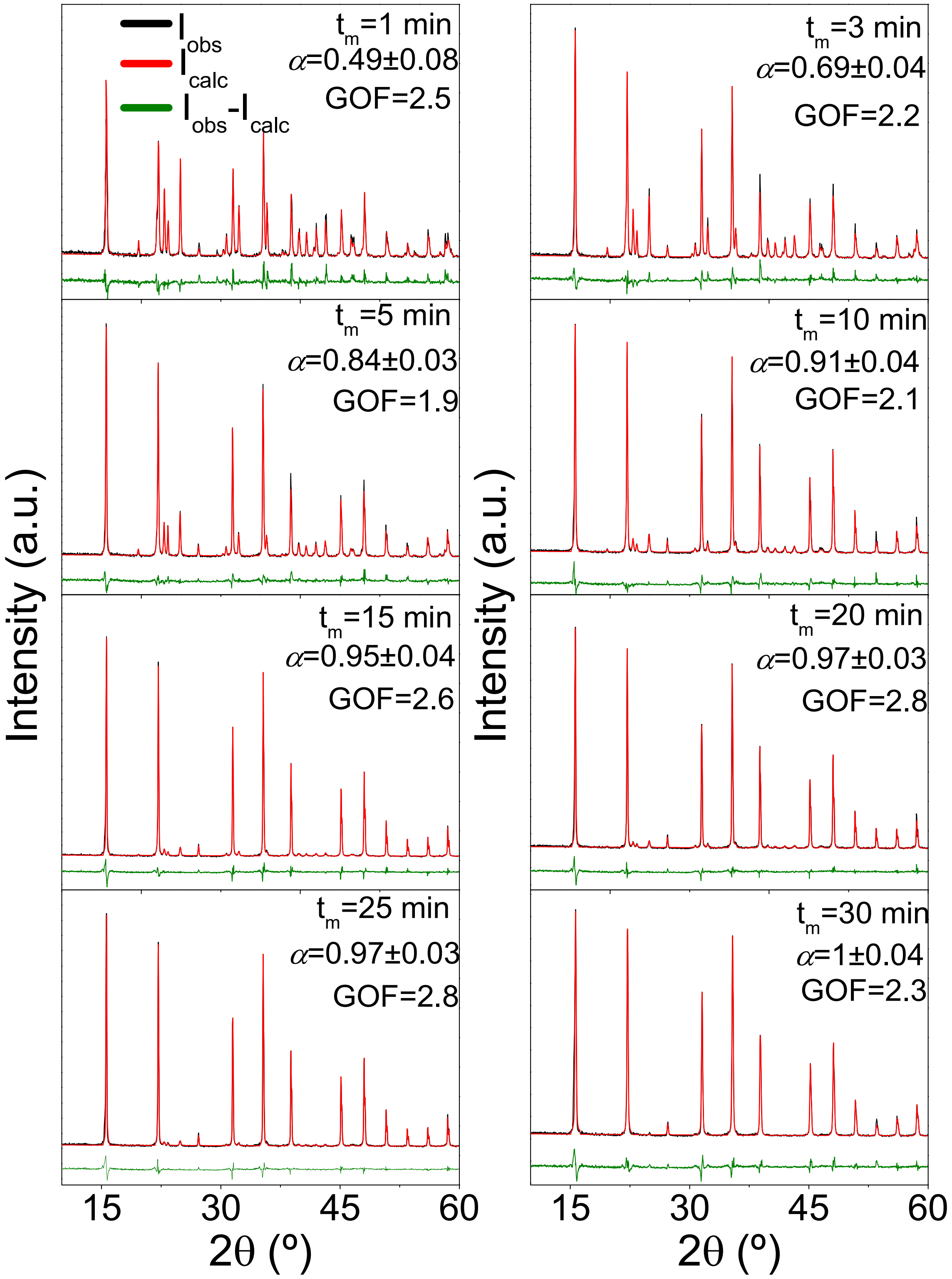

Figure S 7. Rietveld Refinements of selected XRD patterns at different milling times for experimental Condition 5 (P = 9.6 W).

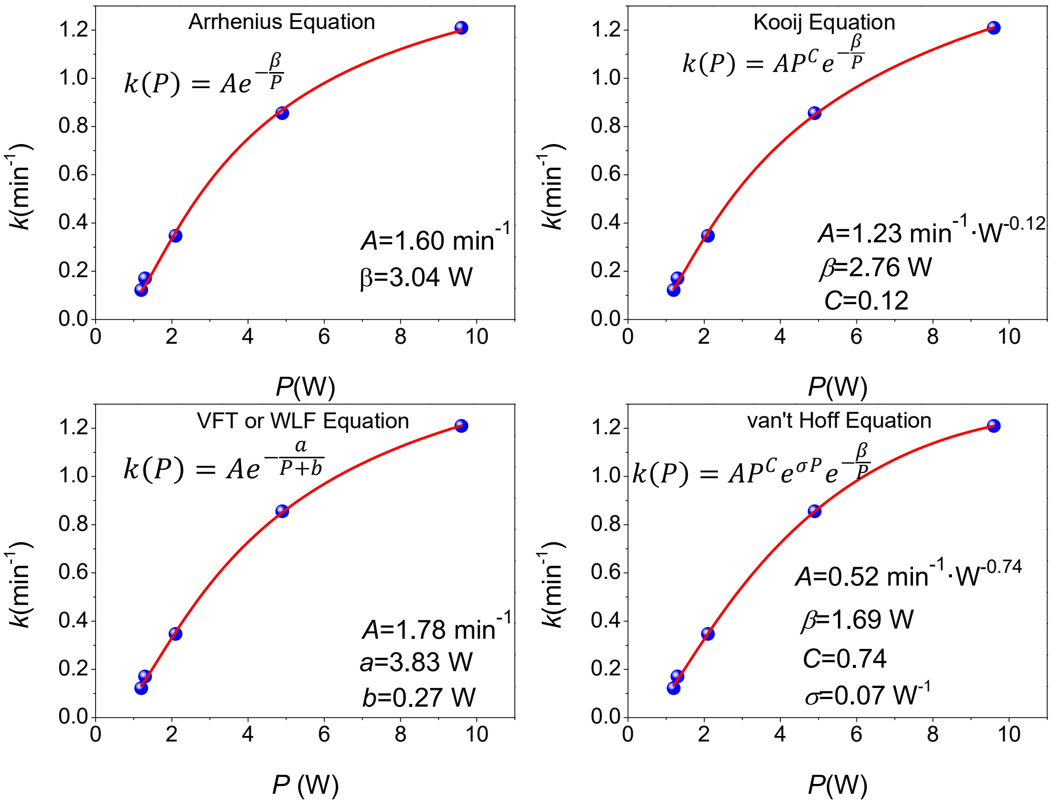

Figure S 8. Rate constant obtained from the v_1 vector as a function of the input power supply and its fittings using the Arrhenius equation as well as several Non-Arrhenian equations (Kooij, VFT or WLF and van't Hoff).

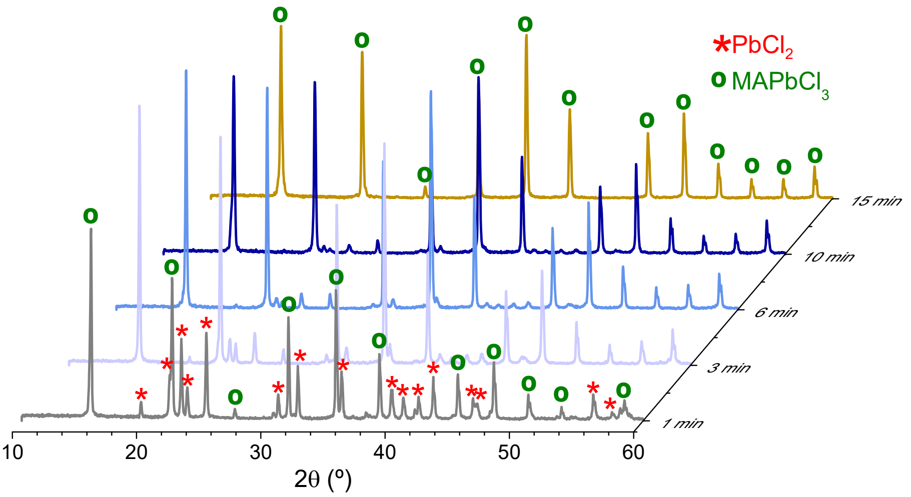

Figure S 9. Selected XRD patterns as a function of the milling time for the milling experiment of Table 2, carried out in a planetary ball mill.

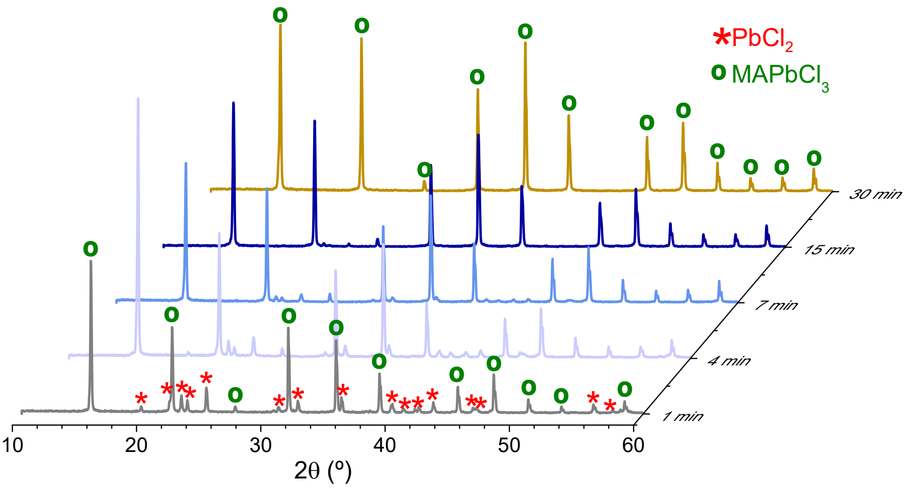

Figure S 10. Selected XRD patterns as a function of the milling time for the milling experiment of Table 2, carried out in a SPEX mill.

5. References

(1) Burgio, N.; Iasonna, A.; Magini, M.; Martelli, S.; Padella, F. Mechanical Alloying of the Fe−Zr System. Correlation between Input Energy and End Products. *Nuovo Cim. D* **1991**, *13* (4), 459–476. https://doi.org/10.1007/BF02452130.

(2) Gil-González, E.; Rodríguez-Laguna, M. D. R.; Sánchez-Jiménez, P. E.; Perejón, A.; Pérez-Maqueda, L. A. Unveiling Mechanochemistry: Kinematic-Kinetic Approach for the Prediction of Mechanically Induced Reactions. *J. Alloys Compd.* **2021**, *866*, 158925. https://doi.org/10.1016/j.jallcom.2021.158925.

(3) Gotor, F. J.; Achimovicova, M.; Real, C.; Balaz, P. Influence of the Milling Parameters on the Mechanical Work Intensity in Planetary Mills. *Powder Technol.* **2013**, *233*, 1–7. https://doi.org/10.1016/j.powtec.2012.08.031.

(4) Chen, W.; Schoenitz, M.; Ward, T. S.; Dave, R. N.; Dreizin, E. L. Numerical Simulation of Mechanical Alloying in a Shaker Mill by Discrete Element Method. *KONA Powder Part. J.* **2005**, *23* (23), 152–162. https://doi.org/https://doi.org/10.14356/kona.2005018.

(5) Concas, A.; Lai, N.; Pisu, M.; Cao, G. Modelling of Comminution Processes in Spex Mixer/Mill. *Chem. Eng. Sci.* **2006**, *61* (11), 3746–3760. https://doi.org/https://doi.org/10.1016/j.ces.2006.01.007.

(6) Muhandis; Nurdiana, F.; Wismogroho, A. S.; Rochman, N. T.; Handoko, L. T. Extracting Physical Observables Using Macroscopic Ensemble in the Spex-mixer/Mill Simulation. *AIP Conf. Proc.* **2009**, *1169* (1), 235–240. https://doi.org/10.1063/1.3243257.

(7) Davis, R. M.; McDermott, B.; Koch, C. C. Mechanical Alloying of Brittle Materials. *Metall. Trans. A* **1988**, *19* (12), 2867–2874. https://doi.org/10.1007/BF02647712.

(8) Maurice, D.; Courtney, T. H. Milling Dynamics: Part II. Dynamics of a SPEX Mill and a One-Dimensional Mill. *Metall. Mater. Trans. A* **1996**, *27* (7), 1973–1979. https://doi.org/10.1007/BF02651946.
